# Signal Enhancement of a Differential Photoacoustic Cell by Connecting the Microphones via Capillaries

**DOI:** 10.3390/s24072105

**Published:** 2024-03-26

**Authors:** Andrey Boyko, Birgit Lange, Sebastian Eckert, Fedor Mayorov, Ralf Brinkmann

**Affiliations:** 1 Institute of Biomedical Optics, University of Luebeck, 23562 Luebeck, Germanyralf.brinkmann@uni-luebeck.de (R.B.); 2 Laboratory of Quantum Optic Technology, Novosibirsk State University, 630090 Novosibirsk, Russia; 3 Laboratory of Laser Molecular Imaging and Machine Learning, Tomsk State University, 634050 Tomsk, Russia; 4 Medical Laser Center Luebeck, 23562 Luebeck, Germany; 5 Hypertech Laser Systems GmbH, 23562 Luebeck, Germanyinfo@hypertech-lasers.de (F.M.)

**Keywords:** photoacoustic spectroscopy, coupled acoustic resonators, gas detection

## Abstract

Differential photoacoustic spectroscopy (DPAS) cells are usually excited on the first longitudinal ring mode, with a microphone situated in the middle of each of the two resonator tubes. However, it is known from other photoacoustic spectroscopy cell designs that connecting the microphones via a capillary can lead to signal enhancement. By means of finite element method (FEM) simulations, we compared such a photoacoustic spectroscopy (PAS) cell with a capillary to a DPAS cell with a capillary attached to each of the two resonators and showed that the behavior of both systems is qualitatively the same: In both the PAS and the DPAS cell, in-phase and anti-phase oscillations of the coupled system (resonator–capillary) can be excited. In the DPAS cell, capillaries of suitable length also increase the pressure signal at the microphones according to the FEM simulations. For different capillary diameters (1.2 mm/1.7 mm/2.2 mm), the respective optimal capillary length (36–37.5 mm) and signal amplification was determined (94%, 70%, 53%). According to the results of these FEM simulations, a significant increase in sensitivity can, therefore, also be achieved in DPAS cells by expanding them with thin tubes leading to the microphones.

## 1. Introduction

Photoacoustic detectors incorporating acoustic resonators are used for gas analysis in the ppm to ppb concentration range. Over the past four decades, photoacoustic detectors have been developed and used in a wide range of applications, including detection of gas leaks in gas-insulated electrical systems and environmental monitoring of greenhouse gases and industrial pollutants [1,2,3]. High sensitivities have been achieved with longitudinally resonant and resonant differential photoacoustic cells [1,4,5,6,7]. The single photoacoustic cell consists of one cylindrical acoustic resonator with open ends, coaxially connected on both sides to larger cylindrical buffer volumes (PAS cell). For fast time response, low acoustic and electric noise characteristics and high sensitivity, a differential Helmholtz resonator was designed, the differential photoacoustic cell (DPAS cell) [1,8]. The DPAS cell considered in our work consists of two parallel cylindrical resonators connected to buffer volumes at the two open ends. The maximum pressure nodes occur at the middle of the cylindrical resonators by exciting the overall DPAS cell at its first longitudinal eigenfrequency. Therefore, a microphone is placed in the middle of each acoustic resonator to obtain maximum signal. At this first longitudinal eigenfrequency, also known as the “ring mode”, the pressure signs on both cylindrical resonators are opposite. By subtracting both microphone signals, one gets the “differential pressure signal.” DPAS cells have been shown to significantly reduce noise caused by heating glass windows and external noise since those pressure variations do have the same sign on both microphones (e.g., [1,9,10]). Sherstov et al. investigated the acoustic modes of several variants of resonant differential Helmholtz resonators—their cell designs used cylindrical channels (Ø9 × 90 mm) and buffer volumes (Ø20 × 8 mm) [9]. With a Laser Photo-Acoustic SF_6_ Gas Analyzer of this design a threshold sensitivity of ~0.1 ppb SF_6_ was reached. The minimum required radiation power of the CO_2_ excitation laser is ~150 mW [11]. To achieve this detection limit with laser sources with lower power, it is necessary to increase the cell sensitivity.

To further increase the sensitivity of a PAS or DPAS cell, one can, for example, reduce the diameter of the resonator cylinders or increase their length [12]. However, the maximum resonator length is limited by the minimum frequency at which the PAS cell is to be operated and the smallest possible resonator diameter is limited by the laser beam diameter. We, therefore, looked for another way to increase the sensitivity of the DPAS cell. In standard PAS as well as DPAS cell designs, the microphones are directly attached to the cylindrical resonator walls. For PAS cells, it has already been shown that connecting the microphone to the cylindrical resonator via a thin capillary tube can lead to signal enhancement [13]. Our work aimed to investigate whether this is also transferable to differential cells. First, we performed finite element simulations (FEM) to investigate the influence of an additional capillary tube of varying length on the eigenfrequencies and pressure signal of a simple PAS system with similar resonator dimension (Ø8 × 90 mm) as the DPAS cell design of Sherstov et al. [9]. The properties of the “simple” PAS cell were then compared with the DPAS cell with capillaries. Simulations for the DPAS cell with varying capillary length of different diameters showed the theoretical maximum signal gain that can be obtained by attaching the microphones via thin tubes.

## 2. Materials and Methods

When gas or fluid molecules are rapidly heated and cooled, the deposition of thermal energy creates acoustic waves in the form of pressure fluctuations. The acoustic pressure p on position r and time t can be determined by solving the inhomogeneous Helmholtz acoustic equation

(1) $$\frac{\partial^{2}p(r,t)}{\partial t^{2}}-v_{s}^{2}\nabla^{2}p(r,t)=\left(\gamma -1\right)\frac{\partial H(r,t)}{\partial t}$$

where $v_{s}$, γ, and H are the speed of sound, the adiabatic coefficient of the gas, and the heat density deposited in the gas (see e.g., [12]). In a PAS cell, the heat density *H* is generated by an intensity modulated laser beam (if the absorption coefficient of the gas is wavelength dependent, wavelength modulation can also be used). The sound propagation strongly depends on the system geometry—if the intensity is modulated with an eigenfrequency of the resonator, a standing wave pattern forms and the signal is amplified according to the quality factor (*Q* factor or *Q* value) of this mode. The *Q* value is the ratio of the energy *E_S_* stored in the resonator oscillating at the frequency *f_0_* to the energy dissipated per cycle $\dot{E}$ [14]

(2) $$Q=2\pi \;f_{0}\frac{E_{S}}{\dot{E}}$$

Alternatively, it is defined as the ratio of the resonance frequency to its bandwidth

(3) $$Q=\frac{f_{0}}{∆f}$$

The first longitudinal resonance frequency of a single cylindrical resonator can be analytically calculated with the equation

(4) $$f_{001}=\frac{v_{s}}{2(L+\Delta L)}\;,$$

where *L* is the cylinder resonator length, $v_{s}$ the speed of sound, and ΔL is the end-correction, which should be added to the length of the cylinder resonator for each open end. The end correction can be approximated by ΔL≅8/(3π)r for a flanged open end of a cylinder with radius *r* [15].

**Figure 1:**
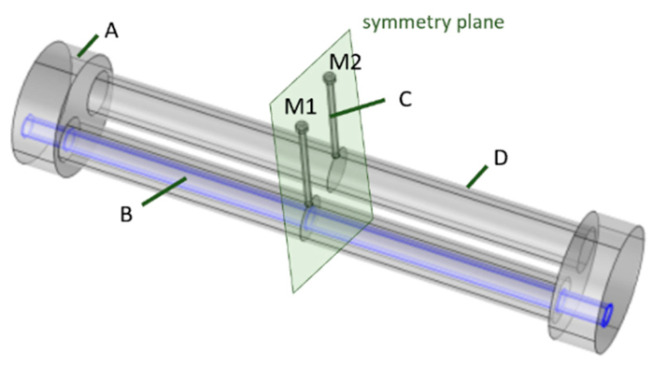
Differential PAS cell geometry setup (FEM simulations). A: buffer volume (Ø22 × 8 mm), B: heat volume, C: capillary tube, D: resonator tube (Ø8 × 90 mm), M1–M2: microphones (not included in the FEM simulation).

Numerical simulations were performed using a finite element method (FEM) software (COMSOL Multiphysics^®^, Version 6.1, COMSOL AB, Stockholm, Sweden; pressure acoustics, frequency domain interface, mesh type “free tetrahedral”, mesh size: “fine”; the simulation settings are also listed in Table 1). For the gas inside the cell, air was selected from the COMSOL material library, and assumed as thermally conducting and viscous in the pressure acoustics fluid model. The “Thermoviscous Boundary Layer Impedance” boundary condition with no slip and isothermal wall was chosen for all boundaries but one: For computational efficiency, the numerical investigations of the DPAS cell are performed with half of the cell geometry by using a “symmetry” boundary condition on the symmetry plane (see Figure 1). The combined effect of all loss mechanisms lead to the resulting quality factor *Q* [16]. Initial settings were a temperature of T = 293.15 K and an absolute pressure of *p_A_* = 1 atm. The PAS cell considered consists of one cylindrical resonator (Ø8 × 90 mm) connected to the microphone via one long capillary with diameter 1.2 mm attached to the main resonator at its middle. The microphone itself is not included in the FEM simulation. The diameter of the buffer volume cylinders is 45 mm, their length was set to 45 mm (half of the resonator length for optimal suppression of window signals, see e.g., [17]; the radius was chosen to get the eigenfrequency of the PAS cell near that of the DPAS cell). The simulated differential PAS cell (DPAS) geometry has two cylindrical resonators (Ø8 × 90 mm, distance of the center axes 11 mm) which are connected at both ends via a buffer volume (Ø11 × 8 mm). One microphone is placed in the middle of each main resonator (not included in the FEM simulations), connected via a long capillary as seen in Figure 1. The simulations were carried out with Ø1.2 mm/1.7 mm/2.2 mm capillaries in view of the later experimental implementation, as glass capillaries of these diameters are available.

In eigenfrequency studies, the resonant modes were determined with COMSOL. Due to the losses, the eigenfrequency values are complex *f = f_r_ + i f_c_* and the *Q* factor can easily be determined by *Q* = *f_r_/2* × *f_c_*. Even though the length of the buffer volumes of the PAS/DPAS cell differ, their first longitudinal eigenfrequencies are about the same (see Section 3). Subsequently, the pressure distribution in the cell is determined for the chosen eigenfrequency by performing a simulation in the frequency domain with the heat source being harmonically modulated with the corresponding eigenfrequency. The heat density *H* deposited by the laser source is implemented in a cylindrical volume (Ø4 × 106 mm) along the axis of the main resonator cylinder (PAS cell)/one of the main resonator cylinders (DPAS cell). For the DPAS cell, the length of the “heat cylinder” covers to overall length of the cell (2 x buffer length + length of the main resonator), for the PA cell with longer buffer volumes, the same length (106 mm) was chosen, and the heat cylinder placed symmetrically around the middle symmetry plane, ending at both sides in the buffer volumes. Assuming a gas concentration of *c_g_* = 1 ppm at standard conditions (1 bar, 25 °C → molecule density *N* = 2.46 × 10^19^ molecules/cm^3^) with an absorption coefficient of *σ* = 4.4 × 10^−17^ cm^2^/molecule (estimation of the absorption coefficient of SF_6_ at 10.55 µm) results for

(5) $$H=\frac{P_{abs}}{V_{heat}}=\;\frac{P\;\sigma \;c_{g}\;NL_{res}\;}{\pi r_{l}^{2}L_{res}}=\frac{P\;\sigma \;c_{g}\;N\;}{\pi r_{l}^{2}}=862\;\frac{W}{m^{3}}\;$$

## 3. Results

**Figure 2:**
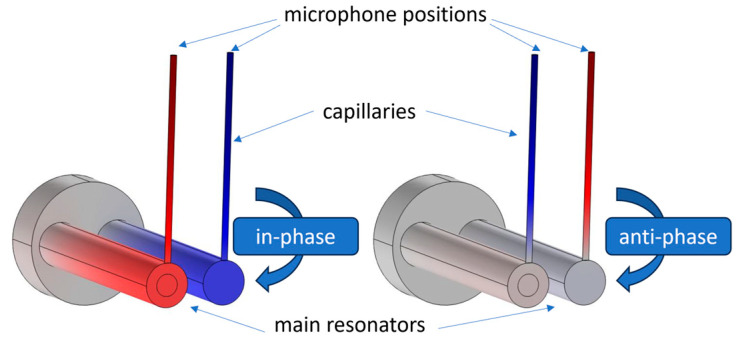
Pressure distribution (at the time point with maximal pressure signal) of the (**left**) in-phase and (**right**) anti-phase oscillation of the first ring mode of the DPAS cell with capillaries connecting the main resonators with the microphones M1 and M2 (not included in the simulation). Note that the highest pressure amplitudes in the “open–closed” capillary are situated at the microphone positions. Length of the capillaries depicted here: 37 mm (increasing their length shifts the pressure node in the capillaries seen on the right side further into the capillaries).

Attaching a capillary to the PAS cell results in a coupled acoustic system that has multiple resonance patterns, even if the uncoupled resonance frequencies of the PAS resonator and the capillary itself are equal [13]. In the coupled system, there is not one first longitudinal eigenfrequency, but two, since there are two types of eigenfrequencies with longitudinal character: “in-phase oscillations” and “anti-phase oscillations”, respectively. In case of the first longitudinal in-phase oscillation at frequency f_1_, the pressure variation of the standing waves in the main resonator and at the end of the capillary (microphone position) do have the same sign. For the anti-phase oscillation with frequency f_2_, the standing waves in the main resonator and the capillary tube oscillate in opposite directions. The pressure distribution of the in-phase and anti-phase oscillation of the first ring mode of the DPAS cell with capillaries connecting the main resonators and microphones are shown in Figure 2. At the time of the largest pressure increase or decrease, the pressure changes in each of the two main resonators and capillaries have the same sign for the in-phase oscillation and the opposite sign for the out-of-phase oscillation, whereas the pressure change has the opposite sign in the two main resonators for both It is important to note that due to the differential measurement, only modes with opposite pressure changes at the microphones are relevant.

**Figure 3:**
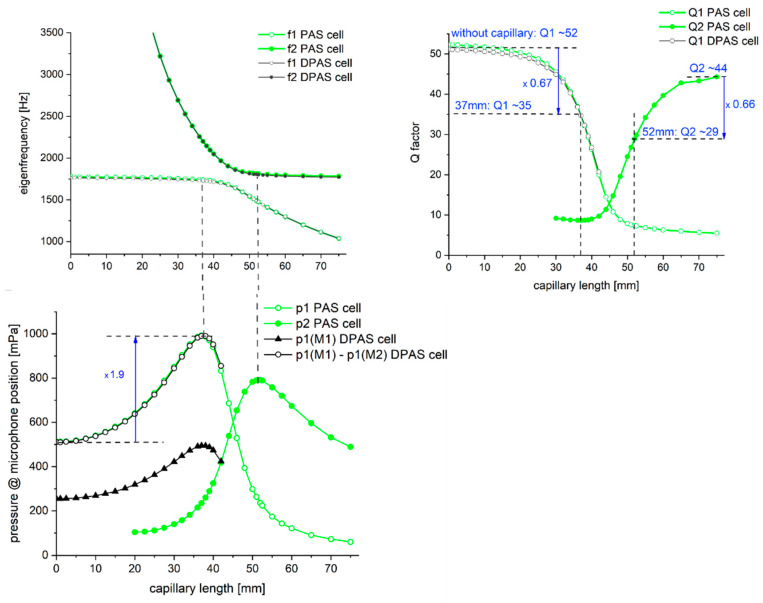
(**Top left**) Eigenfrequencies of the first longitudinal in-phase (f_1_) and anti-phase (f_2_) oscillation of the PAS/DPAS cell with capillary (Ø1.2 mm). (**Bottom**) Pressure signal of the coupled system for the in-phase oscillation (p1) and anti-phase oscillation (p2). For the DPAS cell, one curve shows the pressure signal of the in-phase oscillation at one microphone position (p1(M1), black triangles), the other (black unfilled circles) shows the differential signal of both microphone positions for this mode. (**Top right**) Quality factor of the in-phase (Q1) and anti-phase (Q2) oscillation given by the FEM simulation (green: PAS cell, black: DPAS cell).

In Figure 3 (top left diagram), the frequencies of the first in-phase and anti-phase longitudinal eigenfrequency of the coupled systems are shown for varying capillary lengths (Ø1.2 mm)—the green lines/symbols give the results for the PAS cell, the grey ones are for the DPAS cell. The frequency of the first longitudinal eigenmode of both cell designs is 1775 Hz. The relative deviation of the eigenfrequency values of the PAS compared to the DPAS cell is less than 1%. Attaching a capillary with a length of <30 mm to the main resonator results in a coupled system that can only oscillate in-phase, with a resonance frequency f_1_ of the first longitudinal mode near that of the main resonator itself. Increasing the capillary length leads to a decrease in the Q factor of the in-phase oscillation Q1 (Figure 3, top right). With longer capillary length (≥30 mm), also the anti-phase oscillation can be excited, at a frequency f_2_ higher than that of the in-phase one. For this oscillation, the Q factor increases with increasing capillary length (Figure 3, top right). The deviation of the resonance frequencies of both modes is smallest for a capillary with a length of ~45 mm. In this case, the capillary itself has the same resonance frequency as the main resonator and the Q factor of both oscillations is the same (Figure 3 top right). For capillaries l > 45 mm, the anti-phase mode oscillates near the original frequency of the main resonator whereas the frequency of the in-phase eigenmode is lower, decreasing with increasing capillary length.

The pressure values at the microphone position of the in-phase (Figure 3, bottom, p1, green unfilled circles) and anti-phase oscillation (p2, green filled circles) of the PAS cell are depicted in Figure 3. For the DPAS cell, only the pressure variation of the in-phase oscillation is shown (Figure 3 black triangles: pressure value p1 at one microphone position (M1) of the DPAS cell, black unfilled circles: differential signal, i.e., pressure change p1 at microphone position 1 (M1)—microphone position 2 (M2)). Comparing the absolute value of the pressure amplitude at one microphone only for the in-phase oscillation (p1), this value is higher for the PAS cell than for the DPAS cell due to the bigger overall volume of the latter. However, subtraction of the pressure signal of both microphones of the DPAS cell results in the same signal as that of the PAS cell (with better suppression of external acoustic noise). The curve of the differential pressure signal follows that of the pressure signal of the PAS cell—for both cell designs, one gets the maximal pressure signal for a capillary length of ~36–37.5 mm. For the in-phase oscillation with this capillary length, the quality factor is decreased by ~33% compared to the cells without capillary/ies. For capillary lengths > 42 mm, the pressure amplitudes t both microphone positions of the DPAS cell differ by >3% (up to a factor of >2) in case of the in-phase oscillation (anti-phase oscillation: difference in the pressure amplitude at both microphones <3% for 42 < *l_C_* < 75 mm). For the PAS cell, the anti-phase oscillations show the highest pressure value at the microphone position for a capillary length of ~52 mm (capillary Ø1.2 mm). The overall highest signal increase can be gained by exciting the in-phase oscillation: a signal increase of up to a factor 1.9 is possible attaching the microphone not flush to the main resonator, but via a capillary (Ø1.2 mm × 37 mm). 

**Figure 4:**
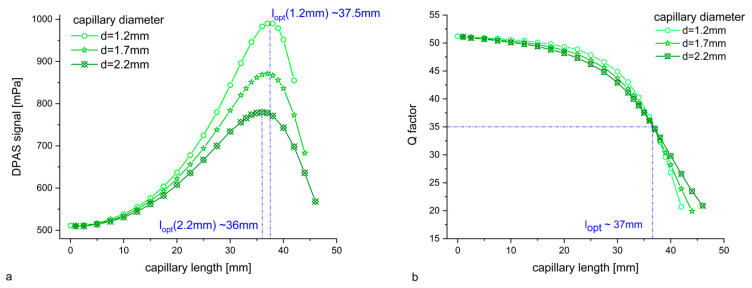
(**a**) Differential pressure signal p1(microphone position 1)–p1(microphone position 2) of the in-phase oscillation of the first longitudinal ring mode for 3 capillary diameters (1.2 mm/1.7 mm/2.2 mm). (**b**) *Q* factors.

For the DPAS geometry, the pressure amplitude p1 of the in-phase oscillation of resonators and capillaries were evaluated for different capillary diameters (1.2 mm/1.7 mm/2.2 mm). Figure 4a shows the “differential signal” p1(microphone position 1)—p1(microphone position 2). For all capillary diameters, the pressure signal increases until a capillary length of 36 mm (Ø2.2 mm) up to 37–37.5 mm (Ø1.2 mm) and decreases for capillaries longer than that. At this point, the *Q* factor is ~35 for all geometries (Figure 4b): as the capillary diameter increases, the resonance frequency for the DPAS cell with a given capillary length decreases. Losses also decrease due the increased volume/surface ratio. Therefore, the *Q* factor stays almost the same. E.g., for a length of 37 mm, the eigenfrequency for the cell with a 1.2 mm diameter capillary is (1726 + 25i) Hz, for a cell with the 2.2 mm diameter one (1659 + 24i) Hz. However, the maximum pressure signal that one can get for the “optimal capillary length” is inversely proportional to the capillary diameter: for Ø2.2 mm, p1(micro 1)–p1(micro 2) is increased by a factor of 1.53 compared to the DPAS cell without capillaries; for Ø 1.7 mm, the FEM simulation predicts a signal increase by a factor of 1.7; and for Ø 1.2 mm, a signal increase by a factor of 1.94.

## 4. Discussion

Trace gas detection using photoacoustic spectroscopy has been a subject of research for several decades [12,18]. Although completely new photoacoustic detectors have been realized, such as Quartz Enhanced Photoacoustic Spectroscopy (QEPAS) [19], in many cases, “classical” acoustic resonators are still used, as described by Miklos and Hess in their review article [1]. Among the systems with the best threshold sensitivites werelongitudinal resonators (cylindrical PAS cells) [20].

Considering the theoretical principle of a PAS gas sensor, there are three ways to enhance its sensitivity [21]: the first one is to increase the laser power, the second one to develop more efficient acoustic transducers (such as tuning forks used in QEPAS), and the third approach is to optimize the geometry of the acoustic resonator to amplify the photoacoustic signal generation. For a longitudinal resonator (PAS cell) driven at its resonance frequency, however, it has been shown that the product of quality factor Q times the cell constant C, which is proportional to the signal amplitude, is about constant [20]. Therefore, the sensitivity can only be increased by simultaneously lowering the Q factor, which happens when you reduce the cylinder radius or increase its length to increase the pressure amplitude at the microphone position. Also, there might be restricting factors such as beam diameter or beam divergence. Additionally, one must consider background signals, which must be minimized in order to lower the detection threshold. Bijnen et al. published in 1996 their work on the geometrical optimization of a longitudinal resonant PAS cell, suppressing window signals by an optimized buffer geometry and tunable air columns [22]. This cell was used in the cavity of a CO_2_ waveguide laser and permitted measurements of ethylene down to 6pptv with a time response of 2 s (trace gas flow 6 l/h). 

Miklos et al. discussed in their paper on the acoustic aspects of photoacoustic signal generation PA detector geometries with the microphone not mounted directly to the resonator but connected by a thin tube [13]. Such constructions have already been applied, e.g., for measuring the PA signal in a hot gas [23]. They showed that in this case, two resonances occur for the coupled system and suggested to exploit the two distinct, strong resonance peaks for simultaneous measurement of two components by two lasers with different wavelengths [13]. They did not, however, discuss the signal increase.

Zha et al. presented in 2022 a “variable diameter T-shaped” cell, which is effectively a cylindrical resonator Ø5 mm × 60 mm with a capillary connecting the microphone that is divided in two parts with different diameters, but a fixed overall length of 10 mm. They optimized this capillary with respect to the pressure amplitude at the microphone position and achieved a signal increase of a factor of 1.6 (capillary Section 1: Ø1 mm × 8 mm, Section 2: Ø5 mm × 20 mm) compared to a cell with a capillary of one diameter [24]. However, this optimization was limited due to the fixed boundary condition on the capillary length. They did not report on the Q factors of the coupled systems.

To investigate the eigenfrequencies of the in-phase and anti-phase oscillation of a coupled system consisting of a “main resonator” Ø8 mm × 90 mm and a capillary of fixed diameter Ø1.2 mm, but varying length, we determined the eigenfrequencies of the first longitudinal modes (pressure variation in the capillary and main resonator in-phase or anti-phase) with FEM simulations. For a capillary length l_c_ smaller than that at which both resonators (“main resonator”: open–open ends and capillary: open–closed end) do have the same eigenfrequency on their own (l_c=r_ ~45 mm), the frequency of the in-phase oscillation of the coupled system is closer to the eigenfrequency of the main resonator. The eigenfrequency of the anti-phase oscillation follows the eigenfrequency of the solitary capillary up to l_c_~40 mm. For capillary length > l_c=r_, the eigenfrequency of the anti-phase oscillation approaches the frequency of the main resonator, while the in-phase oscillation follows the eigenfrequency curve of the capillary. 

The maximum pressure amplitude for a capillary Ø1.2 mm can be obtained for the in-phase oscillation with l_c_~37 mm and for the anti-phase oscillation with l_c_~52 mm. Thus, for both types of coupled modes, the highest pressure increase can be measured for a situation where the eigenfrequency of the coupled system is similar to that of the main resonator cylinder alone. FEM calculations show the pressure amplitude at the microphone position to increase by a factor of 1.94 for the in-phase oscillation (*l_c_* = 37 mm) and only by a factor of 1.54 for the anti-phase oscillation (*l_c_* = 52 mm). The *Q* factor of the in-phase oscillation decreases with increasing capillary length, starting from *Q*~52 (without capillary) to *Q*~35 at *l_c_* = 37 mm. 

To increase the signal to noise ratio (SNR) of a photoacoustic detector, in 1979, a differential setup with two identical, connected Helmholtz resonators was proposed [8]. This approach was further pursued, e.g., by Nordhaus & Pelzl, Zeninari et al. and Song et al. [25,26,27]. In their cell designs, thin tubes were also present, not to connect the “main resonator/s” with a microphone, but to connect the two resonators. In designs for longitudinally resonant differential cells (DPAS), as investigated in the work presented here, the two resonator cylinders are connected via buffer volumes. Sherstov et al. performed an experimental study of the acoustic ring modes that can form in various types of such DPAS cells (as well as single resonator PA cells) [9]. They showed that the ring longitudinal acoustic modes of a differential PAS cell (resonators Ø9 mm × 90 mm, buffer Ø20 mm × 8 mm) do only reach ~1–2 mm into the buffer cavities and, therefore, do not reach the flanges (windows) of the DPAS cell, which significantly reduces background noise. Zheng et al. built a highly sensitive and robust CH_4_ sensor based on a differential PAS design of similar dimensions (two cylinders Ø8 mm × 90 mm, two buffer chambers Ø20 mm × 10 mm) [28]. With a double-pass of a 3.3 µm interband cascade laser beam and an integration time of 90 s, a detection limit of 0.6 ppb was achieved.

The DPAS cell we studied to evaluate if this cell geometry shows the same behavior as the PAS one with respect to the attachment of capillaries has a similar geometry: the two “main resonators” (Ø8 mm × 90 mm) are also connected via the buffer volumes (Ø22 mm × 8 mm). Comparing the eigenfrequencies of the first longitudinal ring modes, in which the main resonators oscillate in anti-phase with respect to each other, and in-phase or anti-phase with respect to the capillaries, one gets good agreement with the results of a PAS cell. Exciting the DPAS cell along one main resonator axis with the same harmonically modulated heat density as the PAS cell results in a lower pressure amplitude at one microphone position due to the bigger cell volume. However, the differential pressure signal (pressure amplitude at microphone 1—pressure amplitude at microphone 2) shows the same behavior with respect to varying capillary length as the PAS cell: for a Ø1.2 mm capillary, the differential signal is a factor of ~1.9 higher for a capillary length of l_c_~37 mm (in-phase oscillation). For an exact analysis of the DPAS system, the pressure values at both microphones must be considered, since certain constellations (long capillaries) show deviations in the pressure increase at both microphone positions. The calculated *Q* factors of both systems’ (PAS/DPAS cell) first longitudinal mode (in-phase oscillation) differ only <3% for *l_c_* < 35 mm and have the same value for *l_c_* = 37 mm (*Q*~35). 

Comparing the FEM results of different capillary diameters (1.2 mm/1.7 mm/2.2 mm) shows that, due to the mainly longitudinal character of the coupled vibration, the optimal capillary length for signal enhancement is similar in the cases considered, as expected (*l_c,opt_* = 36–37.5 mm). The signal increase that can be achieved using the in-phase oscillation is inversely proportional to the capillary radius: 1.2 mm—94%/1.7 mm—70%/2.2 mm—53%. It should be noted that the product of the *Q* factor and the signal amplitude is not constant compared to the DPAS cell without capillaries (*Q_0_*~51, pressure/signal amplitude S_0_: = 1): the decrease of the *Q* factor is ~30% for all diameters, i.e., the product *Q* × S_i_ is increased by a factor of 1.33/1.2/1.05 (Ø1.2 mm/1.7 mm/2.2 mm). Connecting the microphones to the main cells via long, thin tubes, therefore, seems to be a good way of increasing the sensitivity of differential PA detectors. Investigations are planned to verify the FEM results experimentally with a DPAS cell set-up that allows the installation of glass capillaries of different diameters and lengths between the main resonator cells and the microphones.

## 5. Conclusions

According to the results of the FEM simulations, a significant increase in the pressure signal can be achieved for differential PAS cells by connecting the microphones to the resonator cylinders via thin tubes of suitable length. If these simulation results can be confirmed experimentally, the increase in cell sensitivity may open up the possibility of using excitation sources with lower power or reducing the size of the PAS cells without lowering the detection limit.

## Figures and Tables

**Table 1:** Overview of the settings used with the FEM simulations.

Parameter	Setting
Model	Pressure Acoustics, Frequency Domain
Materials	Air
Temperature	293.15 K
Absolute pressure	1 atm
Fluid model	Thermally conducting and viscous
Boundaries	Thermoviscous Boundary Layer
Mechanical condition	No slip
Thermal condition	Isothermal
Mesh	Free tetrahydral (size: fine)

## Data Availability

The data supporting the conclusion of this article will be made available by the authors on request.
